# Pre-transplant imaging evaluation in sickle cell disease: recommended protocols before allogeneic hematopoietic stem cell transplantation

**DOI:** 10.1590/0100-3984.2025.0090

**Published:** 2026-04-23

**Authors:** Fernando da Silva Xavier, Mayara Oliveira da Silva, Luís Henrique Dellabianca Pereira, Adriana Seber, Márcio Luís Duarte

**Affiliations:** 1 United Health Group, São Paulo, SP, Brazil.; 2 Interdisciplinary Health Sciences Program, Universidade Federal de São Paulo, Santos, SP, Brazil.; 3 Universidade de Ribeirão Preto Campus Guarujá, Guarujá, SP, Brazil.; 4 Diagnósticos da América S.A., São Paulo, SP, Brazil.

**Keywords:** Anemia, sickle cell, Stem cell transplantation, Magnetic resonance imaging, Ultrasonography, Doppler, Iron overload, Anemia falciforme, Transplante de células-tronco, Imageamento por ressonância magnética, Ultrassono-grafia Doppler, Sobrecarga de ferro

## Abstract

**Objective:**

To review the recommended imaging modalities for pre-transplant evaluation in patients with sickle cell disease (SCD) undergoing allogeneic hematopoietic stem cell transplantation (HSCT).

**Materials and Methods:**

This was a narrative review focusing on key imaging techniques employed to assess organ damage and stratify risks prior to HSCT in patients with SCD, including transcranial Doppler (TCD) ultrasonography, brain magnetic resonance imaging (MRI) with magnetic resonance angiography (MRA), MRI for osteonecrosis evaluation, and MRI-based hepatic iron quantification.

**Results and discussion:**

TCD, including blind Doppler and TCD imaging (TCDI), plays a critical role in detecting in-creased cerebral blood flow velocities associated with stroke risk. Brain MRI/MRA is essential for identifying silent cerebral infarcts and intracranial vasculopathy, even in patients with normal TCDI velocities. Bone MRI allows early detection of osteonecrosis, which is frequently asymptomatic in SCD. Liver MRI, using R2 relaxometry and T2*-weighting, provides accurate quantification of hepatic iron overload, an important risk factor for transplant-related complications. Cardiac MRI, chest computed tomography, and liver ultrasound are also recommended to help stratify pre-HSCT risk and identify organ damage.

**Conclusion:**

A structured imaging protocol is essential for pre-HSCT assessment in SCD. Radiologists play a pivotal role in identifying subclinical organ damage and providing information critical to transplant candidacy and perioperative management.

## Abstract (pt)

**Objetivo:**

Revisar as modalidades de imagem recomendadas na avaliação pré-transplante de pacientes com doença falciforme (DF) submetidos ao transplante alogênico de célulastronco hematopoéticas (TCTH), destacando seus as-pectos técnicos e relevância clínica.

**Materiais e Métodos:**

Tratase de uma revisão narrativa com foco nas principais técnicas de imagem empregadas para avaliar danos orgânicos e estratificar riscos antes do TCTH em pacientes com doença falciforme (DF), incluindo a ultrassonografia com Doppler transcraniano (DTC), a ressonância magnética (RM) cerebral associada à angiografia por ressonância magnética (ARM), a RM para avaliação de osteonecrose e a quantificação da sobrecarga de ferro hepático por RM.

**Resultados e Discussão:**

O DTC, tanto na forma cega quanto com imagem (DTCI), é fundamental na detecção de velocidades elevadas do fluxo cerebral associadas ao risco de acidente vascular cerebral. A RM cerebral e a ARM permitem identificar infartos cerebrais silenciosos e vasculopatia intracraniana, mesmo em pacientes com DTCI normal. A RM óssea possibilita a detecção precoce de osteonecrose, frequentemente assintomática. A RM hepática, por meio de sequências T2* ou relaxometria R2, fornece quantificação precisa e não invasiva da sobrecarga de ferro hepático, fator relevante para complicações relacionadas ao transplante.

**Conclusão:**

Um protocolo estruturado de imagem é essencial na avaliação pré-TCTH de pacientes com DF, sendo o radiologista peça-chave na identificação de danos subclínicos e no suporte à decisão clínica.

## INTRODUCTION

Sickle cell disease (SCD) is an inherited autosomal recessive hemoglobinopathy that affects approximately 100,000 individuals in the United States and over 3 million people worldwide^**(1)**^. Characterized by chronic hemolytic anemia and recurrent vasoocclusive events, SCD leads to multiorgan damage and reduced life expectancy^**(2,^3^)**^. Although the most common genotype is that of homozygous hemoglobin S, compound heterozygosity with other-globin gene mutations, such as hemoglobin C and -thalassemia, can also produce clinically significant disease manifestations^**(3)**^.

The cumulative morbidity of SCD includes neurological complications such as stroke, silent cerebral infarcts, osteonecrosis, sickle nephropathy, and iron overload due to chronic transfusion therapy^**(4–^8^)**^. These complications contribute to a substantial healthcare burden and increased mortality, especially in adult-hood**(3,^9^)**.

Currently, allogeneic hematopoietic stem cell trans-plantation (HSCT) is the only curative treatment available for SCD, with overall survival rates exceeding 85% and event-free survival approaching 90% in selected pediatric cohorts^**(10)**^. However, HSCT is associated with significant risks, requiring careful patient selection and thorough pre-transplant evaluation^**(5,^8^)**^.

Imaging plays a critical role in that process by detecting organ damage, identifying high-risk patients, and aiding in treatment planning. Essential pre-transplant imaging includes transcranial Doppler (TCD) ultraso-nography, brain magnetic resonance imaging (MRI) with magnetic resonance angiography (MRA), bone MRI for osteonecrosis assessment, and MRI-based quantification of hepatic iron^**(6,^11^–^14^)**^.

This article aims to review the recommended imaging modalities for pre-HSCT evaluation in patients with SCD, emphasizing the technical aspects, diagnostic findings, and the role of the radiologist in guiding clinical decision-making.

## MATERIALS AND METHODS

This is a narrative review of key imaging techniques employed to assess organ damage and stratify risks prior to HSCT in patients with SCD. Searches of the literature were conducted in the PubMed, SciELO, and Embase databases, focusing on publications from 2000 to 2025. Search terms included “sickle cell disease,” “hematopoietic stem cell transplantation,” “magnetic resonance imaging,” “ultrasound,” and “computed tomography.” We performed additional, manual searches of the reference lists of selected articles. Studies were included if they described imaging methods used in the pre-transplant evaluation of patients with SCD.

The results were organized according to imaging modality and clinical purpose, with an emphasis on the main organs evaluated (brain, bones, liver, and heart).

## RESULTS AND DISCUSSION

### Overview

The literature review identified key imaging modalities recommended for the pre-transplant evaluation of patients with SCD undergoing allogeneic HSCT. The most frequently cited techniques were TCD ultrasonography, brain MRI with MRA, bone MRI for osteonecrosis assessment, and liver MRI for iron quantification.

Additional imaging methods—including cardiac MRI, chest computed tomography (CT), and liver ultra-sound—have been consistently mentioned in international guidelines and consensus documents as complementary modalities to evaluate systemic complications and optimize risk stratification prior to HSCT. The findings from these studies are summarized and discussed below, by organ system and imaging modality.

### Imaging for pre-HSCT evaluation in SCD

The clinical indications for allogeneic HSCT in SCD are determined by hematologists on the basis of established international guidelines, considering factors such as stroke history, silent infarcts, organ damage, and treatment failure with other therapies^**(3,^8^,^10^)**^. Once the decision to perform HSCT has been made, imaging plays a fundamental role in assessing disease burden, identifying comorbidities, and stratifying risk before transplantation. This section focuses on the essential imaging modalities recommended for pre-HSCT evaluation in patients with SCD.

### Imaging modalities for evaluating patients with SCD before allogeneic HSCT*r*TCD

#### ultrasonography

For the neurological assessment of patients with SCD undergoing pre-transplant evaluation, TCD ultra-sonography is a fundamental tool because it allows non-invasive measurement of cerebral blood flow velocities, thus providing an early indication of stroke risk^**(6,^12^,^14^)**^. Two main TCD techniques are used in clinical practice: blind TCD (non-imaging Doppler) and TCD imaging (TCDI).

**Figure 1:**
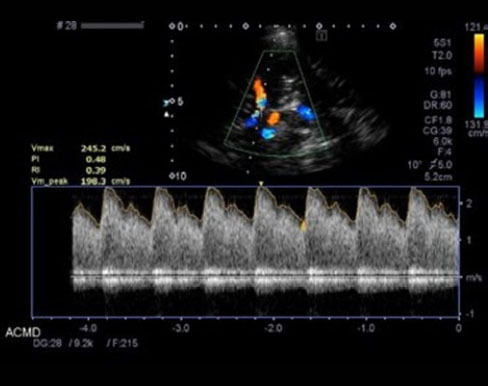
TCD ultrasonography in an adult patient with SCD, demonstrating abnormally increased velocity (TAMMV = 198 cm/s) in the middle cerebral artery, consistent with cerebral hyperemia and elevated stroke risk.

- Blind TCD relies on auditory signals and standard anatomical windows for vessel localization.
- TCDI provides B-mode imaging with color Doppler overlay, allowing direct visualization of intracranial vessels and more precise angle correction^**(12)**^.
- It is important to note that velocity reference values differ between blind TCD and TCDI.
- For blind TCD, according to the criteria established in the Stroke Prevention Trial in Sickle Cell Anemia, an abnormal result is defined as a time-averaged mean maximum velocity (TAMMV) ≥ 200 cm/s in the middle cerebral artery or distal internal carotid artery.
- For TCDI, because of its technical differences (especially in angle correction), the cutoff values are approximately 10–15% lower, with many centers adopting a TAMMV threshold of ≥ 185 cm/s for abnormal classification^**(12,^14^)**^, as illustrated in Figure 1.

The standard insonation protocol involves evaluation of the middle cerebral artery, anterior cerebral artery, posterior cerebral artery, basilar artery, and distal internal carotid artery, using 2-mm stepwise increments along each vessel segment^**(14)**^. In cases of inadequate acoustic windows or technically limited studies, brain MRI with MRA may be necessary for complementary evaluation^**(15–^17^)**^.

Early detection of elevated velocities on TCD or TCDI is crucial, because it directly influences clinical decisions regarding chronic transfusion initiation and the timing of HSCT referral in eligible patients^**(13,^14^)**^.

#### Brain MRI with MRA

Brain MRI combined with MRA plays a crucial role in the pre-transplant evaluation of patients with SCD. These modalities enable detection of both structural brain injuries and cerebrovascular abnormalities that may increase transplant-related risks^**(15,^18^)**^.

**Figure 2:**
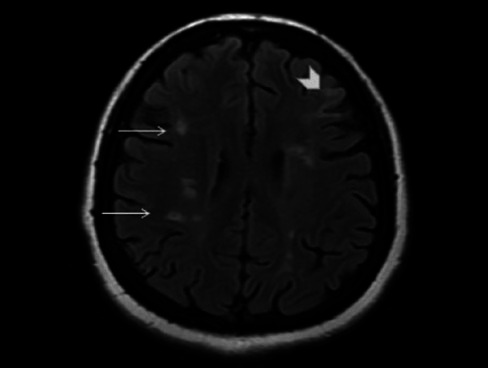
White matter changes in a patient with sickle cell ane-mia. Axial fluid-attenuated inversion recovery MRI at the levels of the centrum semiovale and lateral ventricles showing abnormal increased signal intensity in the periventricular white matter (white arrow) and subcortical white matter (arrowhead).

For identifying symptomatic strokes and silent cerebral infarcts, MRI is the gold standard. Both are common findings in patients with SCD (Figure 2), in whom they are associated with cognitive impairment even in the absence of overt neurological symptoms^**(18–^20^)**^. Silent infarcts are typically defined as lesions that are hyperintense on T2-weighted or fluid-attenuated inversion recovery sequences, measure at least 3 mm at their greatest diameter, are visible in at least two imaging planes, and occur in patients without a clinical history of stroke^**(18,^21^)**^.

**Figure 3:**
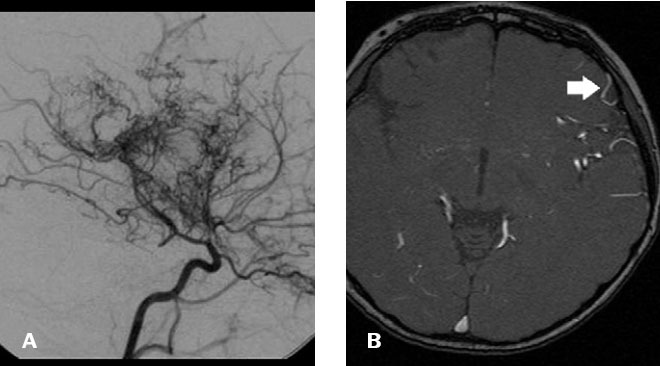
Moyamoya disease in a four-year-old patient. In **A**, digital subtraction angiography image (lateral view) showing extensive collateral vessels from the lenticulostriate circulation and the ophthalmic artery. In **B**, three-dimensional time-of-flight MRI demonstrating an increase in the bilateral lentic-ulostriate flow-void signal due to compensatory collateral circulation (white arrow).

**Figure 4:**
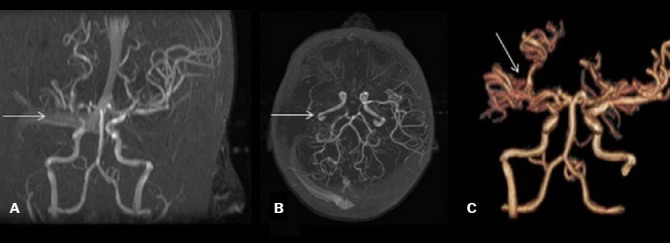
Marked stenosis of the right carotid terminations with a substantial decrease in flow signals of the branches of the right middle cerebral artery (white arrows) and a mild stenosis of the left anterior cerebral artery. Coronal, axial, and three-dimensional views (**A**, **B**, and **C**, respectively).

For evaluating intracranial vasculature, MRA is essential, especially in patients with abnormal or conditional TCD results, or when the acoustic windows are inadequate for reliable Doppler evaluation^**(15–^17^)**^. The use of MRA helps detect intracranial arterial stenosis, which correlates with elevated TCD velocities and can reveal moyamoya vasculopathy, a severe form of chronic cerebrovascular disease commonly associated with SCD^**(^15^,^17^,^22^,^23^)**^, as shown in Figures 3 and 4.

**Figure 5:**
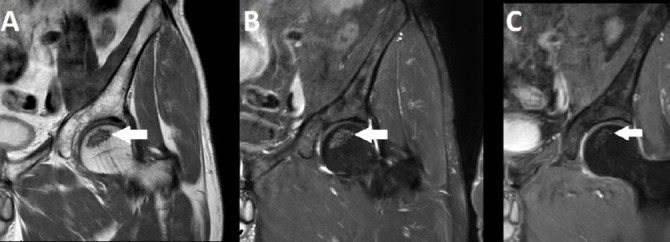
MRI of the left hip. Coronal T1-weighted, proton density-spectral attenuated inversion recovery, and contrast-enhanced T1-weighted spectral presaturation with inversion recovery MRI sequences (**A**, **B**, and **C**, respectively) showing irregular, serpiginous, low-signal lines within the femoral head bone marrow, without contrast enhancement, consistent with osteonecrosis (white arrows).

An important consideration arises from the study conducted by Alshehri et al.^**(24)**^, who employed TCDI to evaluate 86 pediatric patients with SCD. Notably, although all of those patients had normal TCDI velocities (TAMMV < 170 cm/s), silent cerebral infarcts were seen on MRI in 77.8%. This finding highlights the fact that normal TCDI velocities do not exclude the presence of silent infarcts, underscoring the importance of the role that MRI plays in comprehensive neurological assessment, regardless of the TCD results.

Brain MRI and MRA thus provide data complementary to TCD/TCDI, guiding pre-HSCT risk stratification, as well as influencing patient selection and management strategies.

#### Bone infarct detection by MRI

Osteonecrosis is a frequent and disabling musculo-skeletal complication in patients with SCD, affecting up to 30% of young adults, particularly in the femoral and humeral heads^**(25)**^. The pathogenesis is multifactorial, involving microvascular occlusion, chronic ischemia, and bone marrow infarction, and the complication is often asymptomatic in early stages^**(2,^4^,^25^)**^.

The most sensitive imaging modality for early detection of osteonecrosis is MRI (Figure 5), because it allows visualization of bone marrow edema, subchondral fractures, and joint collapse before changes become apparent on conventional radiography^**(25,^26^)**^. Early identification is critical for timely intervention aimed at preserving joint function and delaying disease progression^**(26)**^.

In the pre-HSCT setting, MRI screening is recommended for symptomatic joints or in patients with known risk factors for multifocal osteonecrosis^**(25,^26^)**^. If the patient reports pain in multiple joints or if plain radiographs are inconclusive, whole-joint MRI assessment is indicated^**(25)**^. In addition, studies suggest that once osteonecrosis is diagnosed in one joint, there is a high risk of it developing at additional sites over time, which justifies serial imaging follow-up^**(25)**^.

Furthermore, knowledge of osteonecrotic involvement can influence pre-transplant management strategies, including optimization of physical therapy and orthopedic planning when needed.

#### Quantification of hepatic iron on MRI

Chronic transfusion therapy is frequently needed in the management of SCD, often resulting in progressive iron overload, particularly in the liver^**(5,^27^)**^. Elevated hepatic iron concentration is a recognized risk factor for post-transplant complications, including increased infection risk and liver dysfunction^**(5,^27^,^28^,^29^)**^.

**Figure 6:**
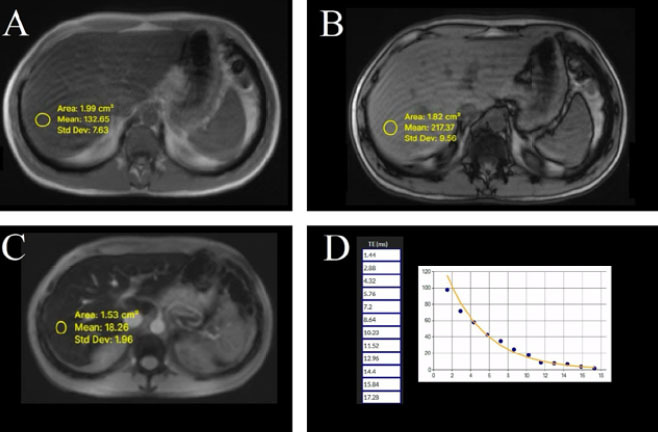
MRI images for hepatic iron quantification in a patient with SCD and chronic transfusional overload. (**A**) Axial in-phase image. (**B**) Axial out-of-phase image demonstrating a relative increase in the hepatic signal compared with the in-phase image, an indirect sign of iron deposition. (**C**) Axial T2* multi-echo image (echo time [TE] = 10.23 ms) showing marked hepatic signal loss, consistent with iron overload. (**D**) Signal decay curve illustrating progressive loss of signal intensity across increasing TEs, confirming the presence of iron overload and allowing quantitative estimation.

Currently, MRI is the preferred noninvasive technique for hepatic iron quantification, with validated methods such as T2* relaxometry, R2 relaxometry (Ferriscan; Resonance Health, Claremont, WA, Australia), and multiecho gradientecho (GRE) sequences for T2* mapping^**(5,^27^,^30^)**^, as depicted in Figure 6.

T2* mapping: provides fast, widely available quantification using standard MRI systems, without the need for contrast agents.

R2 relaxometry (Ferriscan): considered the gold standard for hepatic iron estimation, though it may require specific software and post-processing^**(27)**^.

Multiecho GRE sequences: allow accurate estimation of iron load with short acquisition times, making them suitable for pediatric and critically ill patients when eligible^**(27,^30^)**^.

Given the potential for gadolinium accumulation in iron-overloaded tissues, especially in the liver, contrast-enhanced MRI is generally avoided during hepatic iron quantification^**(5,^30^)**^. Unenhanced techniques remain the standard of care.

Pre-transplant evaluation should include at least one quantitative MRI-based hepatic iron assessment, especially in patients with a history of long-term transfusion or serum ferritin levels exceeding the 1,000–2,000 ng/mL range^**(5,^27^)**^. Hepatic iron concentrations above 7 mg/g dry weight are associated with higher transplant-related risks and may warrant the implementation of iron-reduction strategies prior to HSCT^**(5,^27^)**^.

Regular follow-up with MRI-based quantification is recommended every 12–24 months to monitor treatment response and iron mobilization, especially in patients undergoing phlebotomy or chelation therapy^**(5,^31^,^32^)**^.

#### Cardiac MRI for iron quantification

Chronic transfusion therapy in SCD predisposes to systemic iron overload, with myocardial siderosis being a major cause of morbidity and mortality before HSCT^**(30)**^. Cardiac MRI using T2* relaxometry is the gold-standard noninvasive method for quantifying myocardial iron deposition and guiding chelation therapy^**(33)**^.

**Figure 7:**
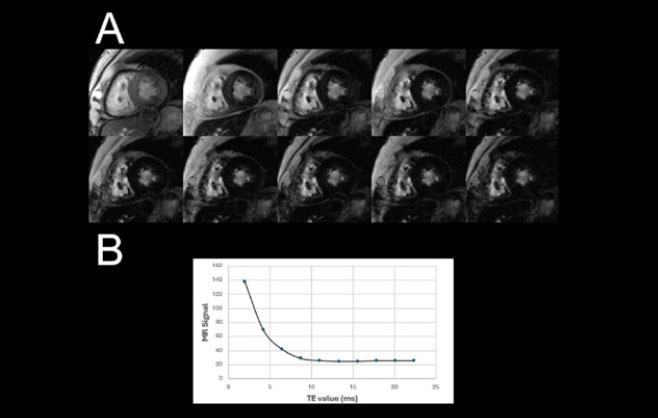
In **A**, Cardiac MRI using a multi-echo GRE (T2*) sequence in a short-axis view, showing progressive myocardial signal loss across ten echo times (TEs) in a patient with severe iron overload. In **B**, corresponding signal decay curve demonstrating a T2* value of 4.2 ms measured in the interventricular septum, confirming marked myocardial iron deposition.

The technique is based on multi-echo GRE sequences that measure the decay of myocardial signal intensity across increasing echo times (Figure 7). As iron content rises, local magnetic-field inhomogeneities shorten T2*, resulting in a diffuse hypointense signal on images of the myocardium. In the landmark study conducted by Anderson et al.^**(34)**^, all patients with ventricular dysfunction exhibited a T2* < 20 ms, and those with values < 10 ms developed overt heart failure. These thresholds remain the main reference for clinical risk stratification.

Myocardial iron shows poor correlation with serum ferritin or hepatic iron, underscoring the need for direct cardiac evaluation. Angelucci^**(33)**^ emphasized MRI-guided assessment as part of standard pre-transplant care to prevent cardiotoxicity. In practice^**(34,^35^)**^, a T2* ≥ 20 ms indicates normal iron load, a T2* of 10–20 ms indicates mild to moderate overload, and a T2* < 10 ms indicates severe siderosis. When the T2* is short, chelation should precede transplantation whenever feasible to minimize peri-HSCT cardiac events.

Beyond iron quantification, cardiac MRI can assess ventricular morphology, function, and fibrosis using cine and late gadolinium enhancement sequences^**(35)**^. Measurement of those parameters improves risk stratification, particularly in patients with longstanding anemia or prior transfusional overload, and provides a comprehensive view of myocardial health before conditioning therapy.

#### Chest CT

**Figure 8:**
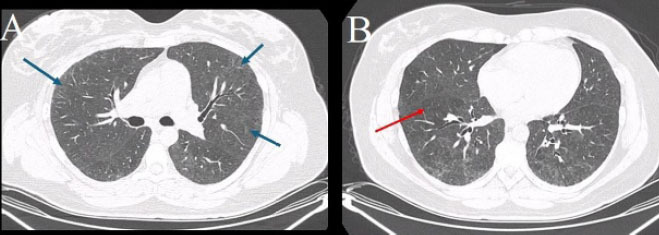
In **A**, axial chest CT demonstrating bilateral ground-glass opacities and interstitial thickening (blue arrows). In **B**, axial chest CT of the same patient showing a mosaic attenuation pattern predominantly in the right lung (red arrow), suggesting ventilation/perfusion mismatch.

Pre-transplant chest CT is valuable for detecting pulmonary sequelae and latent infections that may influence HSCT outcomes. Patients with SCD often show chronic lung injury related to recurrent vaso-occlusive or infectious episodes, with high-resolution CT (HRCT) revealing ground-glass opacities, nodules, mosaic attenuation, or bronchiectasis^**(36)**^, as demonstrated in Figure 8.

In a retrospective study conducted by El Boghdadly et al.^**(36)**^, abnormal CT findings were observed in 48% of asymptomatic pre-HSCT patients, most commonly pulmonary nodules (in 73%) and ground-glass opacities (in 17%), although there was no significant difference between the patients with and without abnormal CT findings in terms of post-transplant pulmonary complications or 100-day mortality. These results suggest that, although CT may uncover subclinical lesions, its prognostic value is limited for routine screening.

Nevertheless, targeted HRCT is recommended for patients with previous acute chest syndrome, chronic hypoxemia, or unexplained pulmonary function decline. The use of HRCT helps rule out occult infection and define prophylactic regimens, as well as facilitating the planning of peri-transplant ventilatory strategies.

When performed pre-HSCT, chest CT also helps quantify the extent of fibrotic or air-trapping changes from prior vaso-occlusive damage, which may predict post-transplant respiratory complications. Integration with pulmonary function testing and echocardiography improves risk assessment for pulmonary hypertension and restrictive lung disease, optimizing transplant readiness.

#### Liver ultrasound

Liver ultrasound is a widely accessible and noninvasive tool for assessing hepatobiliary involvement in patients with SCD before HSCT. Chronic hemolysis, transfusional iron overload, and recurrent inflammation predispose to parenchymal damage, fibrosis, and gallbladder disease. Baseline evaluation helps identify patients at higher risk of hepatic veno-occlusive disease (VOD) and other transplant-related complications^**(37)**^.

**Figure 9:**
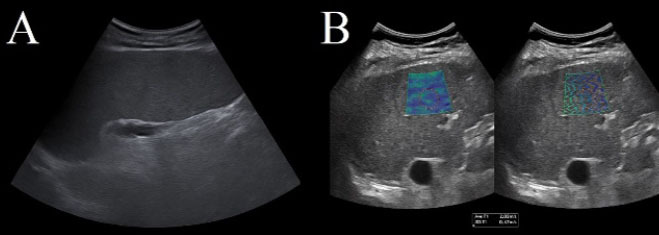
In **A**, Conventional B-mode liver ultrasound showing diffuse textural alteration with increased periportal echogenicity and preserved portal vein flow. In **B**, two-dimensional shear-wave elastography image demonstrating elevated shear-wave velocities (2.03 m/s), indicating increased liver stiffness and suggestive of early fibrosis.

Typical sonographic findings include hepatomegaly, increased echogenicity from hemosiderosis, and, less commonly, focal hypoechoic lesions or gallstones (Figure 9). In a recent study, Adam et al.^**(38)**^ found hepatomegaly in 21.8% of patients with SCD, splenomegaly in 19.3%, and gallstones in 6.4%, highlighting the frequency of chronic hepatosplenic changes. These findings support the use of ultrasound as a sensitive first-line screening tool for structural and biliary abnormalities.

Elastography adds functional value by quantifying hepatic stiffness, aiding in the early detection of fibrosis or congestion related to iron overload or sinusoidal obstruction. When combined with MRI-based iron quantification and determination of serum ferritin, elastography improves the noninvasive monitoring of liver health before HSCT.

In the context of HSCT, Doppler ultrasound is also crucial for detecting early VOD. Lee et al.^**(37)**^ identified pre-transplant hyperferritinemia as an independent risk factor for VOD, with Doppler showing reduced or reversed portal flow in one-third of cases. Therefore, integrating echotexture, flow analysis, and stiffness measurement offers a comprehensive hepatic assessment that can guide preventive measures and reduce transplant-related morbidity.

**Figure 10:**
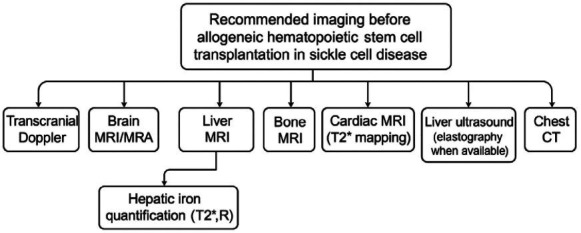
Recommended imaging workflow for pre-transplant evaluation in patients with SCD scheduled to undergo allogeneic HSCT.

The indications for and evaluation of preoperative HSCT imaging tests are shown in Table 1. The imaging workflow is detailed in Figure 10.

**Table 1:** —Summary of indications for and evaluation of pre-HSCT imaging tests.

Imaging modality	Purpose	Recommended technique(s)	Key diagnostic criteria
TCD	Stroke risk stratification	Blind TCD or TCDI	Abnormal TAMMV: ≥ 200 cm/s (blind TCD); or ≥ 185 cm/s (TCDI)
Brain MRI/MRA	Detection of silent infarcts and vasculopathy	T2-weighted, FLAIR, DWI, and TOF MRA	Silent infarcts ≥ 3 mm; arterial stenosis; or Moyamoya changes
Bone MRI	Early detection of osteonecrosis	T1-weighted and STIR sequences of symptomatic joints or screening in high-risk patients	Bone marrow signal alterations suggestive of osteonecrosis
Liver MRI	Quantification of iron overload	T2* relaxometry, R2 relaxometry, or multi-echo GRE sequences	Hepatic iron concentration > 7 mg/g dry weight indicates a high risk of complications
Cardiac MRI for iron quantification	Assessment of myocardial iron overload and cardiac function	T2* mapping sequences (short-axis multi-echo GRE)	T2*: < 20 ms indicates myocardial iron overload; < 10 ms correlates with a high risk of dysfunction
Chest CT	Evaluation of pulmonary infection, fibrosis, or hypertension-related changes	Unenhanced HRCT; contrast-enhanced CT; or CT angiography when clinically indicated	Structural lung changes, ground-glass opacities, bronchiectasis, or thromboembolic findings
Liver ultrasound	Evaluation of hepatomegaly, parenchymal echotexture, the biliary tree, and fibrosis	Conventional B-mode ultrasound; elastography when available	Increased echogenicity suggesting iron overload or steatosis; increased stiffness on elastography indicates fibrosis

FLAIR, fluid-attenuated inversion recovery; DWI, diffusion-weighted imaging; TOF, time-of-flight; STIR, short-tau inversion recovery.

## CONCLUSION

Imaging plays a pivotal role in the pre-transplant evaluation of patients with SCD undergoing allogeneic HSCT. Key imaging modalities—such as TCD ultrasonography, brain MRI with MRA, bone MRI for osteonecrosis assessment, and MRI-based hepatic iron quantification—are essential for identifying organ damage, stratifying risk, and guiding therapeutic decisions.

Early detection of cerebrovascular abnormalities, silent cerebral infarcts, multifocal osteonecrosis, and iron overload allows individualized patient management, optimization of transplant timing, and minimization of peri- and post-transplant complications.

Additional modalities—such as cardiac MRI for myocardial iron quantification, chest CT for the identification of infectious and pulmonary disease, and liver US for structural and elastographic assessment—should be incorporated into the pre-HSCT imaging workflow when clinically indicated. These examinations enhance detection of multiorgan involvement, particularly in transfusion-dependent pediatric patients, and support comprehensive risk assessment.

Radiologists play a central role in multidisciplinary transplant teams, providing crucial diagnostic information that directly influences clinical outcomes in this high-risk population. A standardized multimodality protocol—adapted to patient age and transfusion history—ensures optimal pre-transplant evaluation and improved post-transplant prognosis.

## Data Availability

Not applicable
